# A simplified *in vitro *ligation approach to clone an *E1B55k*-deleted double-targeted conditionally-replicative adenovirus

**DOI:** 10.1186/1743-422X-6-18

**Published:** 2009-02-07

**Authors:** Yosef S Haviv

**Affiliations:** 1 Department of Medicine, Hadassah-Hebrew University Medical Center, Jerusalem, 91120, Israel

## Abstract

**Background:**

Construction of conditionally-replicative Adenovirus (CRAd) is complex and time-consuming. While homologous recombination (HR) using a two-plasmid system in bacteria is commonly used to generate CRAds, alternative methods may be required when HR fails. Previously, *in vitro *ligation has been suggested to facilitate construction of E1/E3-deleted, replication-incompetent Ad vectors. However, *in vitro *ligation has only rarely been used to generate CRAds and may be a complex procedure for molecular biologists who are not experts in the field.

**Methods and Results:**

A modified *in vitro *ligation approach was developed to construct a double-targeted, E1B55k-deleted CRAd. The method allowed the incorporation of a tumor-specific promoter, e.g. the heat-shock protein 70 (hsp70) promoter, upstream of *E1a*, deletion of the *E1B55k *gene, and HR-free cloning of the recombined *E1Δ55k *gene into the Ad genome. The genetic structure of the CRAd was confirmed using restriction analysis and PCR. The replication rate of the hsp70E1Δ55k CRAd was 1.5–2% of Ad without E1Δ55k deletion.

**Conclusion:**

A 3-step cloning approach can generate a double-targeted, *E1B55k*-deleted CRAd using a straight-forward, modified *in vitro *ligation procedure.

## Introduction

Adenoviruses (Ad) are excellent gene transfer vectors and are extensively used for high-level transgene expression *in vitro *and *in vivo*. One of the attributes rendering Ad vectors particularly well suited for this purpose is the capacity to manipulate their genome. Typically, Ad vectors are converted into mammalian gene transfer vectors by replacing the *E1 *gene with the foreign gee of interest. *E1 *deletion serves two goals, i.e. to increase the cloning capacity to 5 kb (and to 8 kb if the *E3 *region is also deleted) and to render Ad vectors replication-incompetent.

Two approaches to have been traditionally used to construct recombinant Ad vectors. First, homologous recombination (HR) in either packaging cell lines, bacteria or yeast. Second, *in vitro *ligation using standard molecular biology procedures. These two methods rely on the fact that purified, linear Ad DNA is infectious, resulting in recombinant Ad virions after transfection into packaging cell lines. *In vitro *ligation was the first approach to manipulate the Ad genome. However, it was abandoned for many years because the large, 35.9 kb Ad genome that encodes more than 50 gene products, generally lacks sufficient unique restriction sites. To overcome this limitation HR was developed, initially using *ClaI *digested viral DNA. Next, the viral DNA was transfected into the packaging cell line to undergo spontaneous, albeit inefficient HR. To enhance HR efficiency in mammalian cells, a two-plasmid rescue system was developed [1,2]. According to this method, two non-infectious plasmids with two overlapping Ad sequences were co-transfected into 293 cells, yielding an infectious recombinant Ad. The first use of the two plasmid system to construct recombinant Ad vectors incorporating foreign transgenes was developed by Graham's lab [3]. The manipulated plasmid encoding the transgene has been termed the shuttle plasmid and was co-transfected into 293 cells with along with a large plasmid containing the Ad backbone, incorporating a 2.2 kb ampicillin resistance gene in the *XbaI *site. Because neither plasmid is infectious alone, recombinant *E1*-deleted Ad vectors were generated as a result of HR between the overlapping regions. Further modifications were made by Bett who removed a large fragment from the *E3 *region and introduced a unique *PacI *site immediately adjacent to the Ad inverted terminal repeat (ITR), allowing linearization of the backbone plasmid [4]. HR thus employs two plasmids with overlapping sequences to be recombined in E1-compelmenting 293 or 911 cells.

To construct Ad vectors encoding heterologous genes, the smaller plasmid, termed the shuttle plasmid and containing the left ITR, a packaging signal and a sequence overlapping with the larger Ad backbone plasmid, was engineered to encode the gene of interest. The larger plasmid, contained almost all the entire Ad genome devoid of the packaging signal and the *E1 *and *E3 *genes, was co-transfected into 293 cells where HR was to generate the recombinant Ad genome. However, this procedure required plaque purification of the recombinant Ad vector by screening individual clones. In addition, HR in 293 cells occurs in low frequency, is a time-consuming procedure and recombinant Ad vector progeny may be contaminated with the wild-type Ad virus. Thus, to obtain a pure Ad preparation at least two rounds of plaque purification assays may be required. To facilitate vector selection in cells, screening for the recombinant virus has been facilitated by using counter-selection methods, extensive fragmentation of Ad DNA complexed with the terminal protein or by using Cre-*lox*-mediated recombination [5]. These methods had the advantage that copies of the recombinant viral DNA were purified from clones and could therefore generate homogenous Ad preparations.

Optimization of two-plasmid HR was further reported in bacterial systems [6], using reconstitution of the sequence of the recombinant Ad in BJ5183 *E. coli *strains before transfection into 293 cells. Another advantage was preparation of the recombinant Ad genome in large quantities before transfection into 293 cells. The BJ5183 bacterial strain is recombination-proficient because i) it lacks the RecBCD enzyme and ii) it contains the RecF enzyme allowing DNA strand exchange between two linear DNA molecules that share at least 50 homologous bp at each end. One of these DNA molecules is the linearized shuttle plasmid previously subcloned with the heterologous gene and containing a bacterial origin of replication, a kanamycin-resistance gene and the left and right inverted terminal repeats (ITR) segments of the Ad genome.

While HR in bacteria has been generally accepted, it requires multiple steps in different *E. coli *stains. In addition, while in mammalian cells, the rate-limiting recombination step produced only the correct viral product of recombination, constructing the Ad genome by recombination in *E. coli *or yeast does not guarantee a correct, infectious recombinant Ad DNA. Unpredicted recombination events can occur, especially in yeast [7], requiring a thorough analysis of the recombinants to exclude clones that are unable to generate virus.

**Figure 1:**
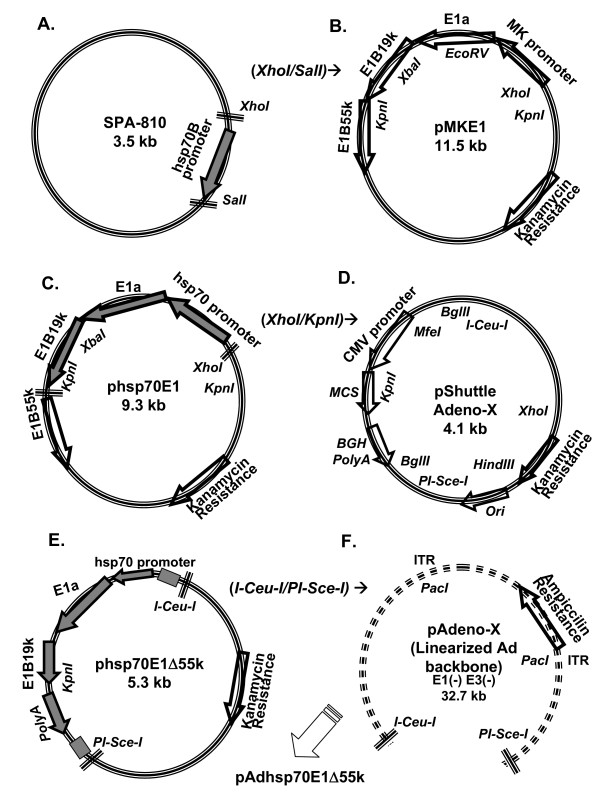
**Overall Schematic diagram of the 3-step cloning strategy to generate CRAd-hsp70E1Δ55k using a modified *in vitro *ligation system**. To generate a double-targeted CRAd, a TSP-regulated E1ΔE1B55k fragment was reintroduced into an E1/E3 deleted Ad backbone. **a**, the 383-bp human hsp70 promoter was cut with *SalI*, Klenow-dNTP blunted, *XhoI *digested, gel purified and inserted into the *EcoRV/XhoI *site in pMKE1 **(b)**, to replace the MK promoter and generate phsp70E1 (**c**). Next, to remove the *E1B55k *fragment, phsp70E1 was digested with *XhoI*, Klenow dNTP blunted and digested with *KpnI *(**c**). Simultaneously, to remove the CMV promoter yet maintain the polyA signal, pShuttle from the Adeno-X system was digested with *MfeI*, Klenow dNTP blunted and digested with *KpnI ***(d)**. Thus, the pShuttle of the AdenoX system was modified to encode a CMV-promoter deleted, TSP-regulated, *E1B55k*-deleted E1 gene in phsp70E1Δ55k (**e**). Next, to generate the recombinant CRAd genome, pAdhsp70E1Δ55k, the hsp70E1Δ55k fragment was removed from phsp70E1 with *I-CeuI *and *PI-Sce-I *digestion, purified via agarose gel electrophoresis and ligated into the pre-*I-CeuI*/*PI-Sce-I*-digested Ad backbone plasmid (**f)**. *SwaI *digestion eliminated the chance of religation of the Ad backbone plasmid without recombination. MK promoter, the human 2.6 kb gene promoter. CMV promoter, the human cytomegalovirus immediate-early gene promoter. BGH polyA, the bovine growth hormone early mRNA poly-adenylation signal. Grey-filled arrows indicate fragments excised for further cloning.

On this basis, *in vitro *ligation would be an attractive alternative procedure to HR. Until a decade ago, *in vitro *ligation was rarely used because of lack of unique restriction sites, low efficiency, the need for plaque purification to exclude wild-type Ad, and the risk of transgene-null Ad vectors due to self-religation. A major breakthrough in the methodology of *in vitro *ligation was the independent introduction of novel unique cleavage sites into the Ad genome by three independent groups [7-10]. Thus, *in vitro *ligation uses whole isolated Ad DNA cleaved with unique restriction sites flanking the E1-deleted region. The digested viral DNA is then ligated directly to a DNA fragment containing the left end joined to the gene of interest, followed by transfection of the recombinant Ad DNA into the packaging cell line [11]. One of the *in vitro *ligation techniques was developed by Mizuguchi and Kay via the introduction of unique intron-encoded endonuclease sites within the Ad genome [8,9] ('AdenoX™'). The *I-CeuI/PI-SceI *intron-encoded endocnucleases uniquely cleave unusually long homing sequences ranging from 15 to 39 bp, rendering these restriction sites rare and ideal for use as cloning sites in large genomes such as Ad. The *E1*-deleted region is flanked by the two unique cleavage sites of *I-CeuI *and *PI-SceI*, and in between a *SwaI *cleavage site is used to inhibit re-ligation of the transgene-null Ad backbone. To produce *E1*-deleted, replication-deficient Ad vectors, this technique requires two cloning steps. First, subcloning the transgene into a *I-CeuI/PI-SceI *flanked, multicloning site (MCS) within the shuttle plasmid upstream of a CMV promoter and downstream of a poly A signal (Fig. 1d). Thus, cutting the recombinant shuttle plasmid with *I-CeuI *and *PI-SceI *provides a full expression cassette based on the foreign ORF cDNA inserted into the MCS. Second, cloning the recombinant *I-CeuI/PI-SceI *fragment from the shuttle vector into the *I-CeuI/PI-SceI *restriction site within the Ad backbone genome. Production of the recombinant Ad genome is thus truly *in vitro *and independent of bacterial or mammalian systems encoding the enzymes essential for HR. However, because the shuttle plasmid contains a CMV promoter upstream of the MCS, tight control of transgene expression may be lost.

Similarly, Souza and Armentano employed the same logic for the construction of Ad serotype 2 vectors using *I-CeuI *and *SnaBI *sites [10]. However, their pAdvantage™ system has a substantially lower reported cloning efficiency than the method reported by Mizuguchi and Kay [8,9].

Danthinne also developed a HR-free system ('AdenoQuick™') for preparation of replication-deficient Ad, using a relatively complex system requiring encapsidation of the recombinant Ad genome into λ phage particles [7].

In the context of oncolytic Ad, re-introduction of modified *E1 *cassettes is required to construct conditionally-replicative Ad (CRAd). The superiority of CRAds over replication-deficient Ad vectors for cancer includes selective amplification of the cancer cell-killing capacity while relatively sparing normal cells. The attributes of CRAds include lysis of tumor cells that are resistant to standard therapy, selective cancer cell killing and induction of cell-mediated anti-tumor immunity. Because there is currently no established effective therapy for disseminated Ad infection, CRAd replication should be stringently restricted to permissive cancer cells. To this end, two genetic approaches have been reported involving either insertion of a tumor-specific promoter (TSP) upstream of *E1a *and *E4 *or partial deletion of *E1 *genes, e.g. *E1B55k *or the *E1A *conserved region 2 (CR2) [12]. Infrequently, double-targeted CRAds may combine both types of genetic modifications.

In contrast to generation of replication-deficient Ad vectors, construction of CRAds is not straight-forward and may be time and labor-consuming. Two molecular strategies to construct CRAds have been described. First, and by far more popular, HR has been used by many groups to recombine an *E1*-encoding shuttle plasmid with the pAdEasy backbone plasmid [13-22]. The second strategy to generate CRAds involves *in vitro *ligation [23-28]. The setbacks of HR in the context of CRAds include the unpredicted chance of *in vivo *HR in BJ5183 bacteria and the lack of sufficient plasmid DNA production in BJ5183 bacteria required to allow diagnostic restriction analysis, thereby requiring transfer to candidate plasmids into other *E. coli *strains, such as DH5α[29].

Despite the utility of HR, potentially targeting virtually the entire Ad genome, when HR fails, alternative approaches to construct CRAds may be required. The two reported approaches to generate CRAds using *in vitro *ligation differ in their methodologies [23-27]. First, Hernandez-Alcoceba et al have reintroduced *E1a *and *E4 *into the Ad genome [23,24]. To generate a CRAd, direct cloning of TSPs upstream of *E1a *or *E4 *within the Ad backbone is feasible using unique restriction sites flanking the promoter regions of E1A (*Bst*BI sites) and E4 (*I-Ce*uI and *Swa*I sites) [23,24].

The setbacks of this approach may include the complete lack of the two *E1B *genes and the large Ad backbone containing the *E1 *and *E4 *genes, rendering manipulation of *E1/E4 *within the large Ad genome less efficient than in smaller shuttle plasmids which are readily amenable for genetic modifications.

The second *in vitro *ligation approach to generate CRAds without HR was developed by Danthinne [25-28]. This method is based on the AdenoQuick™ system, originally developed to construct replication-deficient *E1*-deleted Ad vectors [7]. To construct CRAds, this strategy involves re-introduction of modified *E1 *genes into a shuttle plasmid containing the Ad left arm and the Ad packaging signal. While several CRAds were reported using this system [25-28], the process is rather complex and may require multi-order fragment ligation [25,27] and a *cos *site next to the gene of interest for subsequent *in vitro *packaging into phage λ after cloning the recombinant Ad backbone [7]. Thus, this *in vitro *methodology for CRAd construction may be too labor and time-consuming to investigators who are not experts in the field.

In the current study we report a simplified, HR-free *in vitro *ligation approach to construct a double-targeted, TSP-regulated, ΔE1B55k CRAd involving a direct, 3-step cloning strategy. This modified *in vitro *ligation system is a simple and efficient method to construct recombinant ΔE1B55k CRAds.

## Results and discussion

As a proof of principle to construct a double-targeted CRAd via *in vitro *ligation, CRAd-hsp70E1Δ55k was generated using two genetic modifications, e.g. cloning the heat-shock 70 promoter (hsp70) TSP upstream of the *E1A *gene and deletion of the *E1B55kD *gene (Fig. 1). Overall, we combined components from the AdEasy [6] and the Adeno-X [8,9] systems. An E1-encoding plasmid, constructed on the basis of the pShuttle of the AdEasy system [14], was employed to subclone the hsp70 promoter upstream of E1 (Fig. 1b). However, instead of using this shuttle plasmid for HR, we further subcloned an *E1B55k*-deleted, TSP-regulated *E1 *construct from this shuttle plasmid into the shuttle vector of the Adeno-X system (Fig. 1d). The modified *E1 *construct was then directly cloned into an *E1/E3 *deleted Ad backbone genome using *in vitro *ligation instead of HR (Fig. 1e, f).

**Figure 2:**
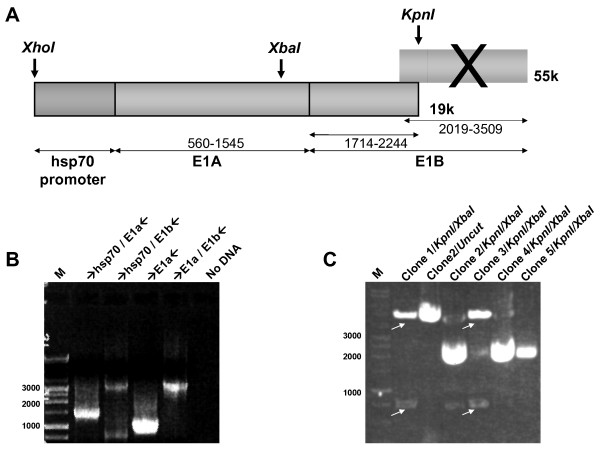
**Validation of hsp70E1Δ55k fragment**. **a**. Schema of the recombinant hsp70E1Δ55k cassette obtained by *XhoI/KpnI *digestion of phsp70E1. **b**. The construct of phsp70E1 (see Fig. 1c) was confirmed by PCR using complementary oligonucleotide amplimers of sequences flanking the indicated fragments of the template plasmid. **c**. Candidate clones for phsp70E1Δ55k (see Fig. 1e) were screened using digestion analysis with *KpnI *and *XbaI*. Diagnostic fragments obtained are marked with an arrow. M-The 1 kb DNA ladder from Gibco was used as DNA size marker.

The first step involved insertion of a TSP upstream of *E1*. The human hsp70 promoter was selected as a TSP because hsp70 expression has been associated with malignancy [30]. Furthermore, heat, ultrasound and magnetic resonance imaging (MRI)-inducible transcription may enable spatial and temporal control of hsp70-regulated gene expression [20,31,32]. Thus, regulation of *E1A *by the *hsp70 *promoter may be a rational TSP approach in the context of CRAds. To insert the 383 bp *hsp70B *promoter (-270→+113) upstream of the *E1A *gene, the SPV-110 plasmid was digested by *SalI*, Klenow-dNTP blunted and cut with *XhoI *(Fig. 1a). Klenow treatment was required to blunt the overhang *SalI *end because the hsp70 promoter was inserted into an *EcoRV*/*XhoI *site within the pMKE1 (Figs. 1b and 2). pMKE1 was originally derived from the shuttle plasmid of the AdEasy system via sequential cloning of *E1 *components into the multiple cloning site [14]. pMKE1 contains the complete *E1 *and protein IX genes and is deleted of the native *E1a *promoter (Δ324–488 nt from the left Ad arm). However, in contrast to the next step of HR in the AdEasy system, the *XhoI/KpnI *digested fragment, encompassing the hsp70 promoter, *E1a *and *E1B19k*, and deleted of the *E1B55k *gene by *KpnI *digestion, was directly subcloned into the pShuttle of the Adeno-X system (Fig. 1d). Within the latter pShuttle, the CMV promoter is retained to drive expression of the heterologous transgenes in replication-deficient Ad vectors [8,9]. However, because CRAds require a TSP upstream of *E1A*, the CMV promoter was excised via *MfeI/KpnI *digestion and replaced by the hsp70E1Δ55k construct, immediately flanked by the stop codons of the AdenoX pShuttle (Figs. 1c–e and 2).

**Figure 3:**
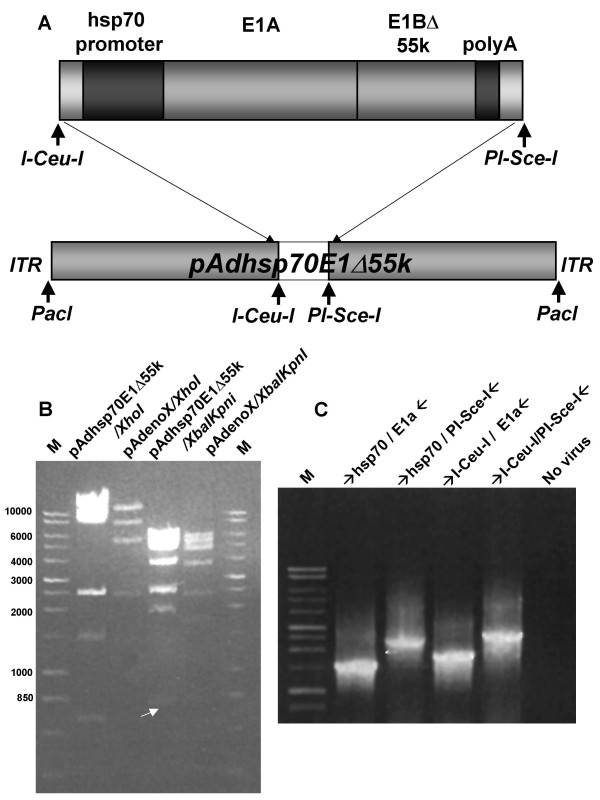
**Validation of pAdhsp70E1Δ55k**. **a**. Schema of the recombinant Ad plasmid pAdhsp70E1Δ55k. **b**. Restriction analysis to confirm the structure of pAdhsp70E1Δ55k by *XbaI/KpnI E1 *cleavage. The diagnostic fragment is marked with an arrow. Control was the Adeno-X Ad backbone plasmid. **c**. Standard PCR was used to confirm the correct structure of the recombinant CRAd-Adhsp70E1Δ55k. The purified recombinant virus was directly applied in the various indicated PCR reactions encompassing the modified E1 gene within the *PI-SceI/I-CeuI *site of the recombinant CRAd.

**Figure 4:**
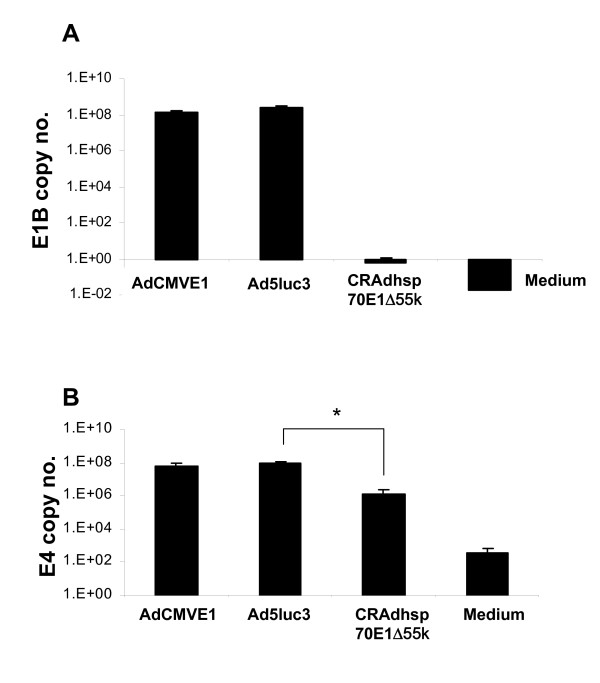
**Analysis of CRAd-hsp70E1Δ55k replication using real-time PCR**. **a**, to confirm E1B55k deletion from CRAd-hsp70E1Δ55k and absence of wild-type Ad, 150,000 A549 cells were plated in 12-well plates and infected after 24-hrs in triplicates with 10 MOI (iu) of CRAd hsp70E1Δ55k, AdCMVE1 or Ad5luc3. Thirty-six hours later cellular DNA was extracted and real-time PCR was performed to measure the E1B gene copy number using primers encompassing the E1B19k (forward primer) and E1B55k (reverse primer) sequences. **b**, to evaluate CRAd-hsp70E1Δ55k replication relative to non-attenuated Ad, real-time PCR was performed on DNA extracted from cells infected above in (**a**), and the E4 gene copy number was measured. *, p < 0.05 for attenuated CRAd-hsp70E1Δ55k replication vs. Ad5luc3 (*t *test).

Next, the *I-Ceu-I/PI-Sce-I *fragment was excised from phsp70E1Δ55k shuttle plasmid, and cloned into the *I-Ceu-I/PI-Sce-I *flanked, *E1*-deletion site within the Adeno-X backbone plasmid to produce pAdhsp70E1Δ55k (Figs. 1e-f, 3). Of note, because the *E1*-deleted Ad backbone pAdeno-X is also deleted of the *E1a *promoter (deleted from nt. 342 of the left arm of the Ad genome), this cloning approach results in a recombinant CRAd genome with TSP-regulated *E1a *expression. This ligation procedure resulted in the correct recombinant CRAd genome in 10% of ampicillin-resistant colonies screened by restriction analysis and confirmed by PCR (Fig. 3). After large scale preparation of the recombinant plasmid with Qiagen Maxi-kit, the recombinant pAdhsp70E1Δ55k plasmid was linearized with *PacI *digestion and transfected into 293 cells to produce CRAd-hsp70E1Δ55k without the need for plaque purification. Absence of wild-type Ad contamination in CRAd-hsp70E1Δ55k stocks was confirmed after three viral passages by absence of *E1B55k *DNA detection via real-time PCR (Fig. 4a). Thus, a double-targeted CRAd was generated using straight-forward HR-free, *in vitro *ligation.

Of note, CRAd-hsp70E1Δ55k manifested a decreased cytopathic effect relative to AdCMVE1 (not shown), deriving from attenuated viral replication. The relative viral DNA replication rate of CRAd-hsp70E1Δ55k after 36 hours was 1.98% and 1.52% of Ad viruses driven by the CMV promoter (AdCMVE1) or the native E1a promoter (Ad5luc3), respectively (Fig. 4b). The fidelity of the hsp70 promoter in CRAd hsp70E1Δ55k was not maintained during heat induction, i.e. there was no heat-shock induction of CRAd hsp70E1Δ55k DNA replication (not shown). This finding is in accordance with previous reports of hsp70 promoter in replicating Ad [20,33]. Thus, while heat-shock enhances the oncolytic effect of CRAds in general [34], the hsp70 promoter does not provide tight control in the context of CRAd-hsp70E1Δ55k.

CRAd-hsp70E1Δ55k differs from two previously-reported CRAds with hsp70 promoters. The first hsp70 CRAd contained a ΔE1B55k deletion, but the hsp70 promoter did not drive *E1a *expression but rather a cytosine deaminase/thymidine kinase (*CD/TK*) suicide gene [33]. The second hsp70 CRAd combined both hsp70 and mouse tyrosinase promoters to drive expression of *E4*, without tumor-targeted partial *E1a *or *E1B *deletions [20]. Of note, both these hsp70-CRAds, as the vast majority of other CRAds, were produced using HR.

Deletion of the *E1B55k *gene was the genetic modification in the first CRAd, *dl*1520 (Onyx-015) [35]. The deletion of a viral gene such as *E1B55k *that inactivates cellular regulatory proteins, e.g. p53, was suggested to restrict CRAd replication to cancer cells with specific genetic mutations thought to complement the viral genetic loss of function [35]. The Ad *E1B *gene encodes two major species of mRNAs. One mRNA codes for a 19-kDa polypeptide (*E1B19k*) and the other codes for a 55-kDa protein (*E1B55k*). The two proteins are encoded by alternative reading frames and share no sequence homology. During Ad infection the E1B proteins inhibit apoptosis to allow Ad protein production and viral DNA replication. While the E1B19k protein has an anti-apoptotic effect mimicking the cellular *bcl-2*, The E1B55k protein exerts its anti-apoptotic effect via inhibition of p53-induced transcription. In addition, E1B55K induces a cellular environment conductive for viral protein synthesis via a complex with the Ad E4 ORF6 protein. This complex inhibits the transport of host cellular mRNA from the nucleus to the cytoplasm while selectively stabilizing and transporting viral mRNA. Because approximately half of human cancer types are mutated for *p53*, deletion of the *E1B55k *gene has been suggested as a tumor-targeted approach suggesting conditional Ad replication only in p53-mutant cells [35]. This approach has been subsequently disputed [36], but *E1B55k *deletion is still considered one of the genetic approaches to mitigate Ad replication in normal cells [12], probably via loss of E1B55k-mediated late viral RNA export rather than p53-inactivation [36]. The cloning strategy reported here allows the deletion of *E1B55k *via digestion of the *E1*-encoding vectors pMKE1 or phsp70E1 at the *kpn*I site. A prior step of subcloning a TSP upstream of the *E1a *gene allows the construction of a double-targeted *E1 *gene for subsequent cloning into the Ad genome. Thus, in view of the relatively complex construction of CRAds using HR [13-22] and previously-reported *in vitro *ligation approaches [23-28], the method we propose has the advantage of only 3 standard cloning steps required to produce a double-targeted CRAd.

The drawbacks of this approach include limitation to *E1*-encoding expression cassettes free of *Cla*I, *Swa*I and *Pac*I, *Bst*BI and *Psp1406I *sites. In addition, *in vitro *ligation is generally limited to recombining the ends of the Ad genome at the *E1 *and *E4 *regions, while HR can use linearized vectors to target virtually any region in the Ad backbone to allow point mutations and small deletions.

In conclusion, HR and *in vitro *ligation have been frequently and rarely employed to generate CRAds, respectively. The simplified *in vitro *ligation system described herein may be efficiently employed to construct TSP-driven, *E1Δ55k*-deleted CRAds.

## Materials and methods

### Cells, plasmids and reagents

293 and A549 cells were from ATCC (Manassas, VA) and maintained in DMEM supplemented with 10% fetal calf serum, 100 units/ml of penicillin and 100 μg/ml of streptomycin in 5%CO2. The SPV-110 plasmid (StressGen, Victoria, Canada) contains the 383 bp human hsp70B promoter (GenBank accession no. X13229), flanked by *XhoI *and *SalI *restriction sites. pMKE1 was previously described [14]. Briefly, it was derived from the shuttle plasmid of the pAdeasy system where the multi-cloning site and right arm in the pAdeasy pShuttle were replaced by the 2.6 kb midkine (MK) promoter and the complete Ad *E1 *region and protein IX genes, devoid of the native Ad E1a promoter region (Δ324–488) [14]. pShuttle and pAdenoX backbone were from Clontech, San Jose, CA (currently BD, Franklin Lakes, NJ). All restriction enzymes and ligase were from New England BioLabs except for *I-CeuI *and *PI-Sce-I *(Clontech). All DNA purificaiotn and cell lysis kits were from Qiagen (Valencia, CA).

### Standard molecular biology procedures for *in vitro *CRAd construction

Screening for recombinants was with miniprep plasmid DNA prepared by standard alkaline lysis kit (Qiagen), restriction analysis and 0.8% agarose gel electrophoresis with ethidium bromide staining. Large scale Ad backbone plasmid and recombinant Ad plasmids were purified by phenol-chloroform extraction and ethanol precipitation or Maxi-prep kit (Qiagen). Digested hsp70 promoter and hsp70E1Δ55k fragments were purified by gel extraction after agarose gel electrophorsesis. To clone the hsp70E1Δ55k fragment from phsp70E1 into pShuttle (Fig. 1c, d), a ratio of 3:1 (insert: digested pShuttle) was optimal. Ligation of the *I-Ceu/I-PI-Sce-I *flanked hsp70E1Δ55k fragment (after digesting 1 μg of phsp70E1Δ55k for 3-hrs) into 750 ng of the pAdeno-X backbone was performed with T4 DNA ligase overnight at 16°C. Cloning efficiency was enhanced by heat inactivation of the T4 DNA ligase and by digesting with *SwaI *that recognizes a unique site between the *I-Ceu-I/PI-Sce-I *sites of the Adeno-X backbone (but not within the *I-Ceu-I/PI-Sce-I *fragment from phsp70E1Δ55k), thereby preventing religation of the *E1*-deleted backbone. The recombinant pAdhsp70E1Δ55k plasmid was used to transform 50 μl chemical-competent DH5α bacteria via 60 sec heat-shock and selection with ampicillin (100 μg/ml).

To rescue CRAd-hsp70E1Δ55k, 5 μg of pAdhsp70E1Δ55k were linearized with *PacI *to expose both ITRs and DNA was purified by phenol-chloroform. Next, to transfect 293 cells, 4 μg of the digested DNA was mixed at room temperature with 20 μl Lipofectamine (Life Technologies), diluted in 500 μl of OptiMEM (Life) and added to the DMEM media for 4-hrs. Media was replaced to growth media and 293 cultured for 12 days until a cytopathic effect was observed. After three cycles of viral propagation, CRAd-hsp70E1Δ55k was purified using double CsCl density gradient centrifugation and tittered using both DNA optical density at 260 nm (viral particle [vp] concentration) and plaque assays in HEK 293 cells (infectious units [iu]). The vp/iu ratio was 100.

### Validation of constructs using PCR

The oligonucleotide primer corresponding to the human hsp70B promoter was sense strand 5'-AGCTAGAACCTTCCCCGCG-3'. The oligonucleotide primers corresponding to the Ad *E1a *were sense strand 5'-TCTTGAGTGCCAGCGAGTAG-3' and the antisense strand was 5'-AAGTCCAAAGGTTGCCCAGG-3'. The oligonucleotide primers corresponding to the Ad *E1B *were sense strand 5'-TTTTCTGCTGTGCGTAACTT-3' and the antisense strand was 5'-ATCTTCATCGCTAGAGCCAA-3. For whole *E1 *PCR the forward primer was the sense sequence of *E1a *and the reverse prime was the antisense sequence of *E1B*. The oligonucleotide primer corresponding to the forward *I-Ceu-I *sense strand was 5'-TAACTATAACGGTCCTAAGGTA-'3 and the reverse *PI-Sce-I *primer was 5'-TTTCTCCGCACCCGACATAGA-'3. To analyze viral DNA with standard PCR, 1 μl of purified CRAd-hsp70E1Δ55k was directly applied into a 50 μl PCR reaction with 1 μl of each primer, at 30 cycles of 95°C 1-min denaturation, 50°C 2-min annealing and 72°C 2.5-min extension.

### Analysis of CRAd replication with real-time PCR

One hundred and fifty thousand A549 cells were infected in triplicates with 10 MOI of CRAd hsp70E1Δ55k, AdCMVE1 or Ad5luc3. Thirty-six hours later (to allow one cycle of complete Ad DNA replication) cellular DNA was extracted using a Blood DNA purification kit (Qiagen). Real-time PCR conditions were 35 cycles of (94°C, 20 s→ 55°C, 20 s → 72°C, 30 s). Ad backbone plasmid pTG3602 (Transgene, Strasbourg, France) was available for plotting a standard curve for the *E4 *copy number. *E4 *copy numbers were normalized to the *β-actin *DNA copy number.

One μl of eluted DNA sample was analyzed by real-time PCR amplification to measure Ad *E4 *copy number as an indicator of CRAd replication and *E1B *copy number to confirm *E1B55k *deletion in CRAd-hsp70E1Δ55k and absence of wild type Ad contamination. The oligonucleotide primers corresponding to the E1B sense strand were 5'-GACAGGGCCTCTCAGATGCT-3' (Ad genome 3075–3094) and the antisense strand was 5'-5'-TGGCTACGTGAATGGTCTTCAG-3' (Ad genome 3144–3123). The oligonucleotide primers corresponding to the E4 sense strand were 5'-TGACACGCATACTCGGAGCTA-3' (Ad genome 34885–34905) and the antisense strand was 5'-TTTGAGCAGCACCTTGCATT-3 (Ad genome 34977–34958). The TaqMAn probe was 5'-CGCCGCCCATGCAACAAGCTT-3' (Ad genome 34930–34951).

## Abbreviations

Ad: Adenovirus; CRAd: Conditionally-Replicative Adenovirus; HR: homologous recombination; MOI: multiplicity of infection; hsp: heat shock protein; MK: midkine; MCS: multiple cloning site; ITR: inverted terminal repeat; TSP: tumor specific promoter.

## Competing interests

The author declares that he has no competing interests.

## Authors' contributions

YSH-designed the cloning strategies, performed the experiments and wrote the manuscript.
